# Sustainable labour market participation among working young adults with diagnosed attention deficit/hyperactivity disorder (ADHD)

**DOI:** 10.1016/j.ssmph.2023.101444

**Published:** 2023-06-12

**Authors:** Magnus Helgesson, Linnea Kjeldgård, Emma Björkenstam, Syed Rahman, Klas Gustafsson, Heidi Taipale, Antti Tanskanen, Lisa Ekselius, Ellenor Mittendorfer-Rutz

**Affiliations:** a Department of Clinical Neuroscience, Division of Insurance Medicine, Karolinska Institutet, SE-17177, Stockholm, Sweden; b Department of Public Health and Caring Sciences, Health Equity and Working Life, Uppsala University, SE-752 37, Uppsala, Sweden; c Department of Medical Sciences, Psychiatry, Uppsala University, Uppsala, Sweden; d Niuvanniemi Hospital, Kuopio, Finland; e School of Pharmacy, University of Eastern Finland, Kuopio, Finland; f Department of Women's and Children's Health, Uppsala University, Uppsala, Sweden

**Keywords:** ADHD, Attention-deficit/hyperactivity disorder, Sequence analysis, Labour market participation, Core-peripheral theory, Sick leave, Disability pension, Social welfare

## Abstract

**Background:**

The aims were to study the sustainability of labour-market participation five years after an incident diagnosis of attention-deficit/hyperactivity disorder (ADHD) among young adults with gainful employment, and to examine the impact of sociodemographic-, work- and health-related factors on these findings.

**Methods:**

Swedish registers identified 2517 individuals, 19–29 years old, with an incident diagnosis of ADHD and gainful employment during 2006–2011. Labour-market participation was measured by the core-peripheral model, a model that measures the connection to the labour market from a weak connection (peripheral) to a strong connection (core). Sequence analysis analysed clusters of labour-market participation, from one year before and up to five years after diagnosis. Odds ratios (OR) with 95% confidence intervals (CI) between sociodemographic factors, comorbid disorders, and the identified clusters were analysed by multinomial logistic regression.

**Results:**

Five clusters of labour-market participation were identified: 60% of individuals belonged to a cluster that maintained labour-market participation throughout the study period (core, close to core); 20% belonged to a cluster with a transition to a weak connection to the labour market (close to peripheral, peripheral); and 20% belonged to a cluster with “middle” labour-market participation, characterised by having long periods of sick leave and unemployment. Individuals with elementary school as highest attained education (OR:4.03;CI:2.35–6.93), comorbid mental disorders (OR:2.77;CI:2.10–3.66), or living in villages/small cities (OR:1.77;CI:1.25–2.51) were most likely to belong to a cluster transitioning towards a “peripheral” labour-market participation. Men were less likely to have peripheral labour-market participation than women (OR:0.55;CI:0.40–0.75).

**Conclusions:**

Over half of working individuals with ADHD maintain a strong attachment to the labour market several years after their first diagnosis of ADHD. Therefore, it is important to target those who have problems maintaining a position in the labour market, including women, those with low educational levels, and those living outside large cities.

## Highlights

• 60 per cent of working young adults with ADHD remains in the labour market five years after an incident diagnosis of ADHD.
• 10 per cent of working young adults with ADHD have an especially high probability to lose their link to the labour market.
• Women, low educational level, living outside large cities and comorbid mental disorders are factors connected to losing work.

## 1 Background

Worldwide, an increasing number of young adults get diagnosed with attention-deficit/hyperactivity disorder (ADHD) at adult age, at a time in life when they are supposed to establish themselves in the labour market (Edvinsson, 2017; Giacobini, Medin, Ahnemark, Russo, & Carlqvist, 2018; National Board of; Health and Welfare, 2014; Rydell, Lundström, Gillberg, Lichtenstein, & Larsson, 2018). Many young adults with ADHD have difficulties finding and maintaining a position in the labour market, and only 30% of them are reported to have gainful work at the time of their ADHD diagnosis (Chen et al., 2021; Kupper et al., 2012). Therefore, it is vital to look more closely at those who have employment to elucidate the factors associated with success, as well as failure, in establishing themselves in the labour market. The results from this study provide insights into which factors enable young adults with ADHD to remain in work. As studies regarding a selected population with ADHD who have gainful work are lacking today, the results of this study can give employers and occupational health staff guidance on how to help these young adults to have a sustainable working life.

When receiving a first (incident) diagnosis of ADHD in adult age, several interventions intended to facilitate both everyday life and work life are introduced. Employers are recommended to take an active part in these programs, giving the workplace a key role in the success of the rehabilitation process (National Board of Health and Welfare, 2014). However, there might not be equal opportunities to adapt work tasks within different sectors, e.g. within the fields of healthcare or teaching it might be difficult to avoid contact with others and to avoid stressful situations. Fields of employment might, therefore, be an interesting factor in the ability to maintain a foothold in the labour market. In addition, several other factors might influence whether individuals diagnosed with ADHD are able to remain in the labour market. Sociodemographic factors such as sex, educational level, age at diagnosis, family composition, place of residence (e.g. city, town, village, etc), and country of birth have been reported to be connected to labour market participation (Edvinsson, Lindstrom, Bingefors, Lewander, & Ekselius, 2013; Gershon & Gershon, 2002; Haukenes, Gjesdal, Rortveit, Riise, & Maeland, 2012). Also, work-related factors such as work sector and sphere, and previous unemployment may affect the relationship between ADHD and subsequent labour market participation (Gémes et al., 2022; Helgesson, Tinghog, Niederkrotenthaler, Saboonchi, & Mittendorfer-Rutz, 2017; Shaw et al., 2014). Moreover, health-related factors such as comorbid disorders and prescribed ADHD medication may also be important factors affecting the ability to remain in the labour market (Chen, Lee, Yeh, & Lin, 2013; Cortese et al., 2018; Giacobini et al., 2018; Helgesson et al., 2017; Kupper et al., 2012; Zetterqvist, Asherson, Halldner, Långström, & Larsson, 2013). Therefore, a programme design that can address many different factors is important for maintaining labour market participation and is warranted to understand the scope of work ability among young adults diagnosed with ADHD. By investigating the association of a variety of factors with the identified clusters of labour market participation, this study can shed light on a range of determinants essential for a sustainable, long-term connection to the labour market for these individuals.

Several models have been used to describe the heterogeneity of labour market participation within a given population. The core-peripheral model operationalises labour market participation as a continuum from attachment (core) to marginalization (peripheral) (Atkinson, 1984). This model has also been developed in a Swedish setting (Gustafsson, Aronsson, Marklund, Wikman, & Floderus, 2014). Together with a sequence analysis model, which is a novel method within epidemiology, the core-peripheral model's main strength is to analyse clusters of labour market participation over time (Kjeldgård, Stigson, Alexanderson, & Friberg, 2020; Murley et al., 2020). By combining sequence analysis with the core-peripheral model, an overview of how labour market participation develops following a diagnosis of ADHD can be achieved.

The aims of the study were to longitudinally follow employed young adults with respect to changes in labour market participation, and to investigate how clusters of labour market participation were associated with sociodemographic, work and health-related factors.

## 2 Methods

### 2.1 Study population

The study base consisted of 8689 young adults aged 19–29 years who were diagnosed with ADHD for the first time in adult age, i.e. after the age of 18 (from *The National Patient Register (NPR)*, administered by the National Board of Health and Welfare, from 1 January 2006 to 31 December 2011). ADHD was defined as having the code F90 in the International Classification of Diseases 10th edition (ICD-10), as the main or secondary diagnosis from inpatient (from 1987) or specialised outpatient (from 2001) healthcare. The day of the incident diagnosis of ADHD in each young adult served as the cohort entry date (CED). In order to include only individuals with a first diagnosis of ADHD, those with a prior diagnosis of ADHD in NPR or with a record of prescribed ADHD medication in the *Prescribed Drug Register (PDR)* from the National Board of Health and Welfare (from July 2005) for codes: N06BA01-N06BA13, and C02AC01-C02AC02 according to Anatomic Therapeutic Chemical Classifications (ATC) before the CED (n = 2668), were excluded. Also, those who emigrated (n = 133) or died (n = 180) during the follow-up period of five years were excluded. Finally, those with no income from work during the year before CED were also excluded (n = 5528). The final study population comprised 2517 young individuals with incident ADHD occurring in young adult age from 2006 to 2011.

### 2.2 Outcome measures

Labour market participation was based on the core-peripheral scale (Atkinson, 1984; Gustafsson et al., 2014), and on annual variables from the *Longitudinal integration database for health insurance and labour market studies (LISA),* administered by Statistics Sweden, one calendar year before and five calendar years after CED, and included five positions in the labour market:

1. Core: in work for most of the year, i.e. time with social benefits (disability pension, sickness absence, municipal support, parental leave, unemployment) or studying not exceeding six months during a year.
2. Close to core: studying or on parental leave for more than six months during a year.
3. Middle: more than 180 gross days of sickness absence or more than 180 days of unemployment during a year.
4. Close to peripheral: municipal support or economic inactivity (which equals no registered income or social benefits in LISA) for more than six months during a year.
5. Peripheral: more than 180 gross days of disability pension during a year.

### 2.3 Covariates

Data linked by the anonymised personal identification number were obtained from the following databases: 1) *LISA* (Ludvigsson, Svedberg, Olen, Bruze, & Neovius, 2019): sex, age at diagnosis, educational level (missing data were regarded as low educational level), family composition, place of residence, country of birth, work sector and sphere (all factors were measured on 31 December the year before CED – categorisation is shown in Table 1); 2) *NPR* (Ludvigsson et al., 2011): inpatient healthcare and specialised outpatient healthcare on comorbid disorders measured as primary or secondary diagnoses in the year before CED, categorised as: mental disorders (ICD-10: F00–F89, F91–F99), somatic disorders (ICD-10: A00-E99, G00-Y99 except O.80); 3) *PDR* (2006–2016) (Wettermark et al., 2007): information of drug prescriptions for somatic disorders (diabetes mellitus (ATC: A10)) and mental disorders (drugs used for addictive disorders (ATC: N07B)), and measured one year before CED; 4) The *Cause of Death Register,* held by the National Board of Health and Welfare (Brooke et al., 2017): date of death (during follow-up).

**Table 1:** Distribution of sociodemographic and medical characteristics in the study population of individuals with incident Attention-deficit-hyperactivity disorder (ADHD, n = 2517).

Sociodemographic factors	N (%)
**Sex**	
Female	1135 (45.1)
Male	1382 (54.9)
***Age at diagnosis***	
19–24 years	1258 (50.0)
25–29 years	1259 (50.0)
***Educational level***^a^	
Elementary (<10 years)	754 (30.0)
High school (10–12 years)	1483 (58.9)
University/college (>12 years)	280 (11.1)
***Country of birth***	
Sweden	2355 (93.6)
Not Sweden	162 (6.4)
***Family composition***^b^	
Married/partnership without children	45 (1.8)
Married/partnership with children	320 (12.7)
Single without children	1994 (79.2)
Single with children	158 (6.3)
***Place of residence***^c^	
Big cities	944 (37.5)
Medium-sized cities	870 (34.6)
Small cities/villages	703 (27.9)
***Work sector***	
Manufacturing	328 (13.0)
Construction	272 (10.8)
Trade and communication	516 (20.5)
Financial and business services	405 (16.1)
Health and social care	478 (19.0)
Other	518 (20.6)
***Work sphere***	
Public sector	2030 (80.7)
Private sector	487 (19.3)
**Medical factors**^d^	
***Comorbid mental disorders***	
No	1345 (53.4)
Yes	1172 (46.6)
***Comorbid somatic disorders***	
No	1354 (53.8)
Yes	1163 (46.2)

a Missing data are coded as low educational level.

b With or without children living at home.

c Place of residence: large cities – Stockholm. Gothenburg and Malmö; medium-sized cities –with more than 90 000 inhabitants within 30 km distance from the centre of the city; small cities/villages – remaining locations.

d All comorbid disorders were measured one year before the cohort entry date. which was the first diagnosis of ADHD in adult age. for depression and bipolar disorder (international classification of diseases. version 10 (ICD-10): F30–F34). anxiety and stress-related disorders (ICD-10: F40–F48). autism-spectrum- disorder (ICD-10: F84). substance abuse (ICD-10: F10–F19 and ATC: N07B). behavioural and emotional disorders (ICD-10: F91–F98). intellectual disabilities/developmental disorders (ICD-10: F70–F83. F85–F89). schizophrenia/psychoses (ICD-10: F20–F29). other mental disorders (all the other codes left starting with F). Somatic disorders included: musculoskeletal disorders (ICD-10: M01-M99). accidents (ICD-10: S00–S99) and other somatic disorders (all other ICD-10 codes except the above-mentioned. O.80 and Z00-99) measured one year before CED.

### 2.4 Statistical analyses

Sequences of annual labour market participation were estimated using sequence analysis during the period beginning one year before and ending five years after the year of the diagnosis Y_0_ (Y_0_ to Y_5_) using TraMineR in R (Gabadinho et al., 2011). The baseline value was measured during the calendar year before the year of the incident diagnosis of ADHD, a year when all included individuals had income from gainful employment.

The distance between sequences was measured using optimal matching with transition matrix costs. Cluster analysis was performed to group similar sequences to identify clusters of labour market participation. Hierarchical cluster analysis using Ward's linkage algorithm and the dissimilarity measures were used (Gabadinho et al., 2011). Thereafter, a sequence index plot of the population was constructed to visualise the heterogeneity in the individual sequences; the sequences are sorted by the state assigned in Y_5_, i.e. the last year of the follow-up period (Supplementary Fig. 1). In addition, a density plot was constructed, illustrating the density of each labour market position every year for all clusters.

The association between sociodemographic factors (sex, age at diagnosis, educational level, family composition, place of residence, and country of birth), work-related factors (work sector and sphere) and comorbid somatic and mental disorders and different clusters of labour market integration were analysed using Multinomial logistic regression. Crude and multivariate-adjusted odds ratios (OR), with 95% confidence intervals (CI) and Nagelkerke r^2^-values, were calculated for these associations. All statistical analyses were performed by R (version 3.5.0).

## 3 Results

A total of 2517 young adults with an incident diagnosis of ADHD in adult age and having gainful work at the time of diagnosis were included in the study. Of those, about 55% were men and 45% were women (Table 1). About 30% had an elementary school education, 59% had a high school education and 11% had a university/college education as the highest attained educational level. There was a fairly even distribution with respect to place of residence (with 38% living in large cities, 35% in medium-sized cities, and 28% living in small cities/villages. Most of participants worked in the private sector, with healthcare being the most common work sector. About 47% had a comorbid mental disorder and about 46% had a comorbid somatic disorder.

### 3.1 Identified clusters

The cluster analysis revealed five different clusters of labour market position (measures supporting the choice of clusters are available in Supplementary Table 1). The distribution of characteristics, as well as the associations between the characteristics and the distribution of the clusters compared to the reference cluster (cluster 1), are found in Table 2.^1^ In the density plots, the distribution of sequences of annual outcomes of the five labour market positions was visualised in the five clusters revealed (Fig. 1). Moreover, a detailed description of the most frequent sequences within each cluster is presented (Supplementary Fig. 2).

**Table 2:** Distribution and adjusted odds ratio (OR) for different factors in each of the five clusters of labour market marginalization status/year over one year before and five years after ADHD diagnosis (Y-1 to Y_+5_) among 2.517 individuals aged 20–29 years.

Total	Cluster 1^a^	Cluster 2		Cluster 3		Cluster 4		Cluster 5	
	n (%)	n (%)	OR (95% CI)	n (%)	OR (95% CI)	n (%)	OR (95% CI)	n (%)	OR (95% CI)
	**1332 (52.9)**	**199 (7.9)**		**502 (19.9)**		**209 (8.3)**		**275 (10.9)**	
**Sex**									
Female	601 (45.1)	106 (53.3)	ref	186 (37.1)	ref	79 (37.8)	ref	163 (59.3)	ref
Male	731 (54.9)	93 (46.7)	0.86 (0.60–1.23)	316 (62.9)	1.28 (0.98–1.66)	130 (62.2)	1.33 (0.93–1.90)	112 (40.7)	**0.55 (0.40**–**0.75)**
**Age at diagnosis**									
19–24 years	681 (51.1)	117 (58.8)	ref	202 (40.2)	ref	107 (51.2)	ref	151 (54.9)	ref
25–29 years	651 (48.9)	82 (41.2)	**0.70 (0.50**–**0.97)**	300 (59.8)	**1.74 (1.39**–**2.18)**	102 (48.8)	1.08 (0.79–1.47)	124 (45.1)	1.06 (0.80–1.41)
**Educational level**									
Elementary	310 (23.3)	63 (31.7)	1.69 (0.98–2.91)	184 (36.7)	**4.38 (2.74**–**7.02)**	92 (44.0)	**2.85 (1.65**–**4.90)**	105 (38.2)	**4.03 (2.35**–**6.93)**
High school	831 (62.4)	113 (56.8)	1.08 (0.66–1.79)	292 (58.2)	**2.72 (1.74**–**4.26)**	97 (46.4)	1.16 (0.69–1.97)	150 (54.5)	**2.01 (1.20**–**3.37)**
University/college	191 (14.3)	23 (11.6)	ref	26 (5.2)	ref	20 (9.6)	ref	20 (7.3)	ref
**Country of birth**									
Sweden	1261 (94.7)	183 (92.0)	ref	463 (92.2)	ref	196 (93.8)	ref	252 (91.6)	ref
Not Sweden	71 (5.3)	16 (8.0)	1.59 (0.89–2.82)	39 7.8)	**1.54 (1.01**–**2.35)**	13 (6.2)	1.20 (0.64–2.24)	23 (8.4)	**1.79 (1.07**–**3.00)**
**Family composition**^b^									
Married/partnership without children	27 (2.0)	5 (2.5)	1.37 (0.51–3.72)	7 (1.4)	0.65 (0.27–1.55)	1 (0.5)	0.24 (0.03–1.81)	5 (1.8)	0.71 (0.26–1.95)
Married/partnership with children	182 (13.7)	26 (13.1)	1.07 (0.67–1.72)	64 (12.7)	0.75 (0.54–1.04)	24 (11.5)	0.78 (0.49–1.26)	24 (8.7)	**0.52 (0.32**–**0.84)**
Single without children	1052 (79.0)	148 (74.4)	ref	393 (78.3)	ref	174 (83.3)	ref	227 (82.5)	ref
Single with children	71 (5.3)	20 (10.1)	**1.95 (1.09**–**3.48)**	38 (7.6)	1.28 (0.81–2.02)	10 (4.8)	0.91 (0.44–1.89)	19 (6.9)	0.81 (0.46–1.43)
**Place of residence**^c^									
Large cities	542 (40.7)	68 (34.2)	ref	160 (31.9)	ref	86 (41.1)	ref	88 (32.0)	ref
Medium-sized cities	451 (33.9)	78 (39.2)	1.37 (0.96–1.96)	175 (34.9)	**1.33 (1.03**–**1.73)**	68 (32.5)	0.99 (0.70–1.41)	98 (35.6)	**1.47 (1.06**–**2.04)**
Small cities/villages	339 (25.5)	53 (26.6)	1.20 (0.80–1.80)	167 (33.3)	**1.60 (1.22**–**2.10)**	55 (26.3)	1.02 (0.70–1.49)	89 (32.4)	**1.77 (1.25**–**2.51)**
**Work sector**									
Manufacturing	162 (12.2)	21 (10.6)	1.01 (0.57–1.82)	74 (14.7)	1.13 (0.77–1.64)	30 (14.4)	1.18 (0.70–1.99)	41 (14.9)	1.46 (0.90–2.35)
Construction	147 (11.9)	20 (10.1)	1.09 (0.60–1.98)	73 (14.5)	1.21 (0.83–1.78)	20 (9.6)	0.84 (0.46–1.5)	12 (4.4)	0.52 (0.26–1.04)
Trade and communication	293 (22.0)	37 (18.6)	ref	95 (18.9)	ref	41 (19.6)	ref	50 (18.2)	ref
Financial and business services	212 (15.9)	29 (14.6)	1.09 (0.64–1.83)	76 (15.1)	1.18 (0.82–1.69)	38 (18.2)	1.36 (0.84–2.21)	50 (18.2)	1.51 (0.97–2.35)
Health and social care	268 (20.1)	44 (22.1)	0.84 (0.47–1.53)	75 (14.9)	0.72 (0.46–1.13)	31 (14.8)	1.15 (0.63–2.10)	60 (21.8)	1.12 (0.67–1.88)
Other	250 (18.8)	48 (24.1)	1.27 (0.76–2.10)	109 (21.7)	1.31 (0.91–1.87)	49 (23.4)	**1.62 (1.00**–**2.61)**	62 (22.5)	1.34 (0.86–2.08)
**Work sphere**									
Public sector	1080 (81.1)	147 (73.9)	ref	405 (80.7)	ref	178 (85.2)	ref	220 (80.0)	ref
Private sector	252 (18.9)	52 (26.1)	1.53 (0.96–2.46)	97 (19.3)	1.40 (0.97–2.01)	31 (14.8)	0.86 (0.52–1.43)	55 (20.0)	0.97 (0.63–1.50)
**Comorbid mental disorders**^d^									
No	818 (61.4)	99 (49.7)	ref	229 (45.6)	ref	102 (48.8)	ref	97 (35.3)	ref
Yes	514 (38.6)	100 (50.3)	**1.56 (1.15**–**2.11)**	273 (54.4)	**1.90 (1.53**–**2.36)**	107 (51.2)	**1.69 (1.25**–**2.28)**	178 (64.7)	**2.77 (2.10**–**3.66)**
Comorbid somatic disorders									
No	765 (57.4)	189 (92.0)	ref	247 (49.2)	ref	115 (55.0)	ref	135 (49.1)	ref
Yes	567 (42.6)	10 (107.0)	**1.42 (1.04**–**1.93)**	255 (50.8)	**1.36 (1.10**–**1.69)**	94 (45.0)	1.09 (0.81–1.48)	140 (50.9)	1.20 (0.91–1.57)

a Cluster 1 was the reference cluster and consisted of individuals with a mainly “core” labour market position throughout the study period. They were more likely to be men, had college/university education, were born in Sweden, living in a single household without children, and only a few had a comorbid mental disorder at baseline.

b With or without children living at home.

c Place of residence: large cities – Stockholm. Gothenburg and Malmö; medium-sized cities – cities with more than 90 000 inhabitants within 30 km distance from the centre of the city; small cities/villages – remaining areas of living.

d All comorbid disorders were measured one year before the cohort entry date. which was the first diagnosis of ADHD in an adult age. for depression and bipolar disorder (international classification of diseases. version 10 (ICD-10): F30–F34). anxiety and stress-related disorders (ICD-10: F40–F48). autism-spectrum- disorder (ICD-10: F84). substance abuse (ICD-10: F10–F19 and ATC: N07B). behavioural and emotional disorders (ICD-10: F91–F98). intellectual disabilities/developmental disorders (ICD-10: F70–F83. F85–F89). schizophrenia/psychoses (ICD-10: F20–F29). other mental disorders (all the other codes left starting with F). Somatic disorders included: musculoskeletal disorders (ICD-10: M01-M99). accidents (ICD-10: S00–S99) and other somatic disorders (all other ICD-10 codes except the above-mentioned. O.80 and Z00-99) measured one year before CED.

**Fig. 1:**
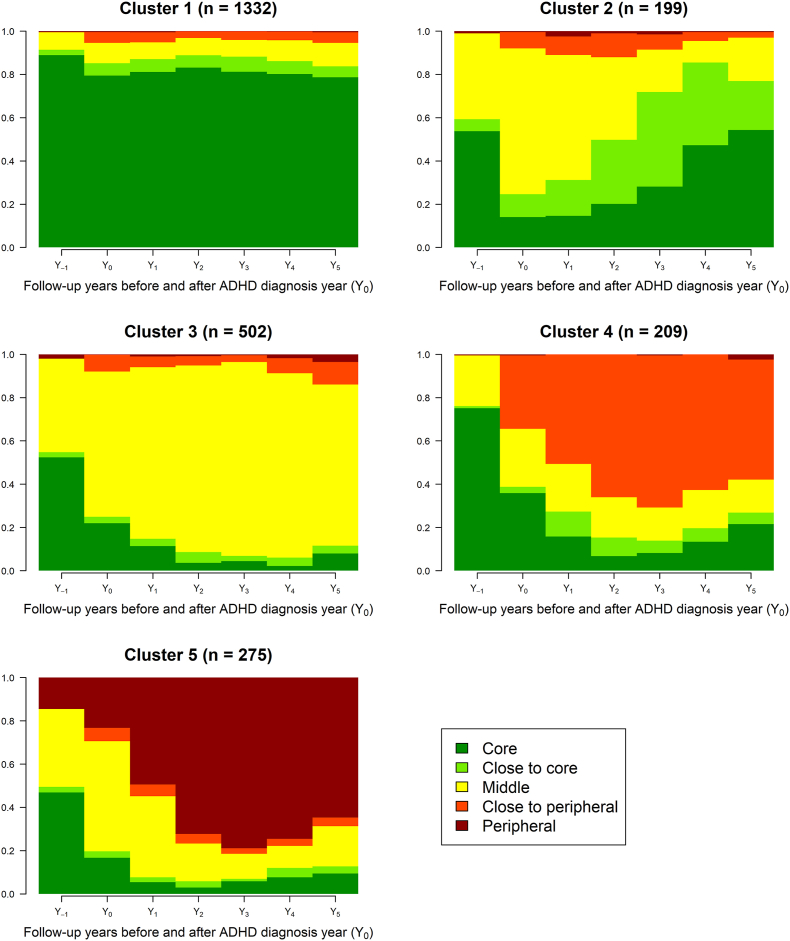
Clusters of annual labour market participation among young adults with attention-deficit/hyperactivity disorder (ADHD) (N = 2517).

Individuals in cluster 1 (53% of the study population, reference cluster), had mainly a core labour market position throughout the study period (Fig. 1). Individuals in cluster 1 were more likely to be men, had attained a college/university educational level, were born in Sweden, were living in a single household without children, and in this cluster, few had a comorbid mental disorder (Table 1).

Individuals in cluster 2 (8%) had, to a large extent, a middle position in the labour market when they received a diagnosis of ADHD. Towards the end of the study period, they had mainly a core or close to core labour market position. Individuals in cluster 2 were more likely to be single with children living at home or had a comorbid mental or somatic disorder.

Similar to individuals in cluster 2, many individuals in cluster 3 (20%) also had a middle position around the time of the diagnosis of ADHD. The difference in comparison to cluster 2 was that those belonging to cluster 3 seemed to remain in a middle labour market position throughout the study period. Individuals in cluster 3 were more likely to be men, had an elementary or high school education, lived in small cities/villages or had mental or somatic comorbidity.

Individuals in cluster 4 (8%) had a transition to a mainly close to peripheral labour market position five years after the diagnosis of ADHD. Individuals in cluster 4 were more likely to have elementary school as the highest attained education level or had mental comorbidities. As in cluster 3, there was also a tendency that men had a higher risk of belonging to this cluster.

Most individuals in cluster 5 (11%) had a transition to a peripheral labour market position during the follow-up period. Individuals in cluster 5 were more likely to be women, to have elementary school or high school as the highest attained education level, to be living in medium-sized cities or small cities/villages, to be born outside Sweden, or to have mental comorbidities.

## 4 Discussion

### 4.1 Main findings

The sequence analysis identified five clusters of labour market participation among young adults diagnosed with ADHD for the first time in adult age. A maintained labour market position, measured as a core or a close to core, was found among about 60% of our cohort, while the rest belonged to clusters with a less stable labour market position. Most of them (20% of the total population) followed the cluster of an increasing middle labour market position, characterised by long periods of unemployment or sickness absence. About 11% belonged to a cluster with an increasing close to peripheral labour market position, characterised by dependence on municipal support or having no record of either work income or income from any social insurance. The remaining 8% belonged to a cluster with an increasing peripheral labour market position, characterised by the granting of disability pension and, most often, permanent exit from the labour market. Individuals with elementary school as the highest attained educational level and those with comorbid mental or somatic disorders were more likely to belong to a cluster with a transition towards a peripheral labour market position. In addition, women were also more likely to follow clusters transitioning towards a peripheral labour market position. There were relatively few differences between work sectors, but those in industry and construction were more likely to belong to a cluster with a middle labour market position, with long periods of sick leave or unemployment.

### 4.2 Identified clusters

Approximately 60% of all included young adults had a strong labour market connection five years after they were diagnosed with ADHD. Compared to a general sample of young adults diagnosed with ADHD, where only about 17% belong to a core labour market position, this is rather a high share and is an indication of a selected group of ‛healthier’ individuals with ADHD in this study. Our results indicate that those managing to stay at work most likely have functional impairment prerequisites that are met by their employers or work situation. It has been reported that, with the right prerequisites being met, many individuals with ADHD can be as productive as their peers without ADHD (Robbins, 2017).

The remaining 40% evolved towards a weaker labour market position during the years after their diagnosis of ADHD and were, to various degrees, dependent on e.g. welfare payments or family. Around 25% of those, equal to 8% of the total sample, were granted disability pension during the follow-up period. It has been reported that only one out of 10 who are granted a disability pension as a young adult will re-enter the labour market (Swedish Social Insurance Agency, 2012). Therefore, measures to enhance labour market participation must be put in place long before disability pension is granted. Two reviews concluded that both Cognitive Behavioural Therapy (Mongia & Hechtman, 2012) and Mindfulness-based intervention (Lee et al., 2017) improve both attention and functioning among adults with ADHD. There are, however, very few studies that also assess if there is a connection between increased functionality and an increase in labour market participation.

Regarding disorders other than ADHD leading to functional disability, studies have reported that without support, preferably support where the employer also takes an active role, it will be far more difficult to return to work (Heijbel, Josephson, Jensen, & Vingård, 2005; Holmlund et al., 2022). In Sweden, according to the guidelines from the National Board of Health and Welfare, interventions are supposed to be given at the time of the first ADHD diagnosis, intended to increase both functional capacity and, among those who have remaining work ability, involvement from a potential employer. Both employers and stakeholders, such as the Swedish Social Insurance Agency and the Swedish Employment Agency, thus have economic incitements to avoid young adults with remaining work ability leaving the labour force permanently.

### 4.3 Characteristics of clusters

For those in clusters with a high proportion of middle labour market position at the beginning of the follow-up period, it is obvious that educational level was a decisive factor for the possibility of maintaining the labour market position. In Sweden, most work will require at least upper secondary school education. Those in cluster 3, i.e. maintaining a middle position throughout the follow-up period, were foremost individuals with only elementary school education. Therefore, many young adults with ADHD did not have the means to acquire sufficient education and are hence extremely vulnerable in the labour market. Also, many young adults with common mental disorders, i.e. depression, anxiety, and stress disorders) have difficulty finding stable work, although not as high a percentage as young adults with ADHD. For those in cluster 2, i.e. a mainly middle labour market position at the beginning of the follow-up, but with a transition to a mainly close to core and core position, educational level seemed less important. The low educational level was also characteristic of those having a close to peripheral or peripheral labour market position. Other studies have also reported the importance of education among young adults with ADHD (Helgesson, Björkenstam, et al., 2021), but this study can add that educational level seems also to be of importance for the ability to remain in the labour market.

Individuals with comorbid mental disorders were more likely found in clusters with a peripheral labour market position. This has been reported also by other studies, and comorbid mental disorders seem to be decisive for later labour market position among individuals with ADHD (Helgesson, Björkenstam, et al., 2021; Helgesson, Rahman, et al., 2021). Person-based treatment and rehabilitation are essential to improve labour market outcomes for individuals with ADHD and mental comorbidity.

Finally, there were some important differences between men and women regarding labour market participation. Men were more represented in clusters of a mainly middle labour market position, while women, to a greater extent, were found in clusters with a mainly peripheral labour market position. In previous studies regarding a general population diagnosed with ADHD, there have been no large differences between men and women regarding labour market participation (Chen et al., 2021; Chen et al., 2023; Helgesson, Björkenstam, et al., 2021; Helgesson, Rahman, et al., 2021). Studies have, however, reported substantial differences between men and women in the symptomatic expression of ADHD (Gershon & Gershon, 2002). This may indicate that symptomatic differences, i.e. that women have more internalised problems than men, might affect the possibility of staying permanently in the labour market (Edvinsson et al., 2013; Gershon & Gershon, 2002). The differences in symptoms between women and men might also lead to fewer women getting an ADHD diagnosis and thus those women who are diagnosed with it have a more severe form (Gershon & Gershon, 2002). Women have also, in general, more spells of work disability compared to men (Allebeck & Mastekaasa, 2004). All these above-mentioned reasons might explain the differences between the sexes.

### 4.4 Strengths and limitations

The utilisation of several longitudinal population-based registers was the main strength of this study. The population-based approach allowed the sequence analysis to be accompanied by powerful and robust clusters and categorisations. Also, the use of a wide range of covariates, including important sociodemographic factors, work-related factors, and medical factors provided vast information regarding the pattern of labour market participation among young adults with ADHD. The results may be used as a base for creating targeted rehabilitation measures for this group.

Also, there are some limitations worth mentioning. ADHD almost always has an onset during childhood and, due to the lack of retrospective data for the years before 2001 (70–75% coverage in 2001–2004, 97% coverage from 2005 (National Board of Health and Welfare, 2020)), we cannot rule out that some individuals in the database had already had a diagnosis of ADHD in specialised outpatient care during childhood. Therefore, we excluded all individuals with a record of prescribed ADHD-related medication before CED. Further, information on sickness absence was accessible only for spells longer than 14 days. As the measure of labour market position, however, was based on more than six months in each state, we regard this as a minor problem regarding study design. We assessed work sectors only at baseline and thus there may have been sector changes during follow-up. A study design able to consider changes in employers may thus give a more accurate result. To have a five-year follow-up period for all individuals at the time of the start of the study in 2019, we assessed individuals from 2006 to 2011 up to 2016. There might have been changes in the treatment of ADHD, as well as in the sickness insurance scheme, during the period of this study.

## 5 Conclusions

The study showed that over half of working individuals with ADHD maintain a strong link to the labour market five years after the first diagnosis of ADHD. Therefore, it is important to target those who are struggling to maintain a position in the labour market, including women, those with a low level of education, and those living outside large cities. The findings of this study can hence give guidance to employers and occupational health professionals on how to optimise a sustainable working life for young adults diagnosed with ADHD.

## Author statement

**Magnus Helgesson:** Conceptualization, Methodology, Validation, Formal analysis, Writing - Original Draft, Writing - Review & Editing, Supervision, Project administration, Funding acquisition.

**Linnea Kjeldgård:** Methodology, Software, Validation, Formal analysis, Data Curation, Writing - Original Draft, Writing - Review & Editing, Visualization **Emma Björkenstam:** Conceptualization, Methodology, Writing - Review & Editing **Syed Rahman:** Conceptualization, Methodology, Writing - Review & Editing **Klas Gustafsson:** Conceptualization, Methodology, Writing - Review & Editing **Heidi Taipale:** Conceptualization, Methodology, Writing - Review & Editing **Antti Tanskanen:** Conceptualization, Methodology, Writing - Review & Editing **Lisa Ekselius:** Conceptualization, Methodology, Writing - Review & Editing **Ellenor Mittendorfer-Rutz:** Conceptualization, Methodology, Resources, Data Curation, Writing - Review & Editing, Supervision.

## Ethics approval

Ethics approval for this project was obtained from the Regional Ethical Review Board of Stockholm, Sweden.

## Funding

This project was supported by 10.13039/501100002706AFA Insurance Agency (grant number 180295), and we utilised data from the REWHARD consortium supported by the 10.13039/501100004359Swedish Research Council (grant number 2017-00624).

## Ethics approval

Ethics approval for this project was obtained from the Regional Ethical Review Board of Stockholm, Sweden.

## Declaration of competing interest

None declared.

## Figures and Tables

**figs1:**
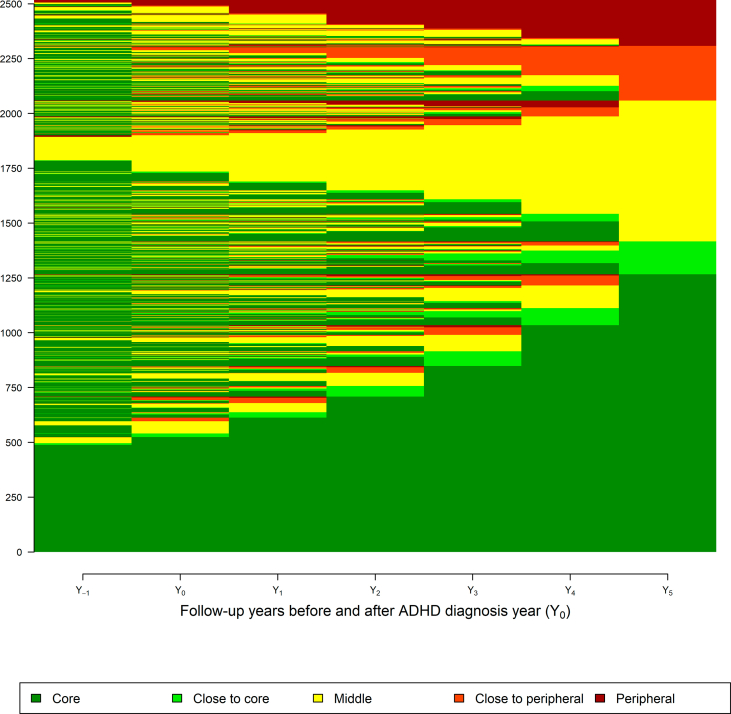
All individual sequences of labour market participation.

**figs2:**
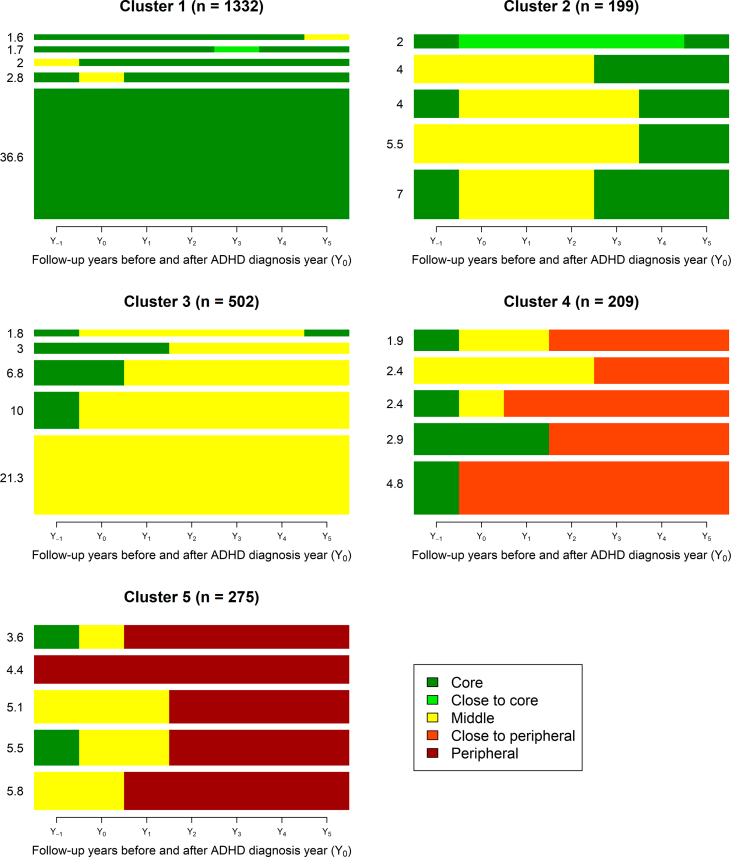
The five most frequent individual sequences of labour market participation within each cluster.

## Data Availability

The authors do not have permission to share data.

## Footnotes

1 The Nagelkerke R^2^ value for the full model was 0.139, meaning that almost 14% of the variance was explained by the included covariates. Variables which explained most of the variance (R^2^ difference between full model and R^2^ of the model where the mentioned covariate was excluded from the analysis exceeded 0.01) were in decreasing order: educational level (0.033), comorbid mental disorder (0.028), age at diagnosis (0.013), sector (0.012) and sex (0.010).

## References

[bib1] Allebeck P., Mastekaasa A. (2004). Chapter 5. Risk factors for sick leave - general studies. Scandinavian Journal of Public Health.

[bib2] Atkinson J. (1984). Manpower strategies for flexible organisations. Personnel Management.

[bib3] Brooke H.L., Talbäck M., Hörnblad J., Johansson L.A., Ludvigsson J.F., Druid H. (2017). The Swedish cause of death register. European Journal of Epidemiology.

[bib4] Chen H.-J., Lee Y.-J., Yeh G.C., Lin H.-C. (2013). Association of attention-deficit/hyperactivity disorder with diabetes: A population-based study [population study]. Pediatric Research.

[bib5] Chen L., Mittendorfer-Rutz E., Björkenstam E., Rahman S., Gustafsson K., Taipale H. (2021). Risk factors for disability pension among young adults diagnosed with attention-deficit hyperactivity disorder (ADHD) in adulthood. Journal of Attention Disorders.

[bib6] Chen L., Mittendorfer-Rutz E., Björkenstam E., Rahman S., Gustafsson K., Taipale H. (2023). Labour market integration among young adults diagnosed with attention-deficit/hyperactivity disorder (ADHD) at working age. Psychological Medicine,.

[bib7] Cortese S., Sun S., Zhang J., Sharma E., Chang Z., Kuja-Halkola R. (2018). Association between attention deficit hyperactivity disorder and asthma: A systematic review and meta-analysis and a Swedish population-based study. The Lancet Psychiatry.

[bib8] Edvinsson D. (2017).

[bib9] Edvinsson D., Lindstrom E., Bingefors K., Lewander T., Ekselius L. (2013). Gender differences of axis I and II comorbidity in subjects diagnosed with attention-deficit hyperactivity disorder as adults. Acta Neuropsychiatrica.

[bib10] Gabadinho A., Ritschard G., Muller N.S., Studer M. (2011). Analyzing and visualizing state sequences in R with TraMineR. Journal of Statistical Software.

[bib11] Gémes K., Björkenstam E., Rahman S., Gustafsson K., Taipale H., Tanskanen A. (2022). Occupational branch and labor market marginalization among young employees with adult onset of attention deficit hyperactivity disorder-A population-based matched cohort study. International Journal of Environmental Research and Public Health.

[bib12] Gershon J., Gershon J. (2002). A meta-analytic review of gender differences in ADHD. Journal of Attention Disorders.

[bib13] Giacobini M., Medin E., Ahnemark E., Russo L.J., Carlqvist P. (2018). Prevalence, patient characteristics, and pharmacological treatment of children, adolescents, and adults diagnosed with ADHD in Sweden. Journal of Attention Disorders.

[bib14] Gustafsson K., Aronsson G., Marklund S., Wikman A., Floderus B. (2014). Peripheral labour market position and risk of disability pension: A prospective population-based study. BMJ Open.

[bib15] Haukenes I., Gjesdal S., Rortveit G., Riise T., Maeland J.G. (2012). Women's higher likelihood of disability pension: The role of health, family and work. A 5-7 years follow-up of the hordaland health study. BMC Public Health.

[bib16] Heijbel B., Josephson M., Jensen I., Vingård E. (2005). Employer, insurance, and health system response to long-term sick leave in the public sector: Policy implications. Journal of Occupational Rehabilitation.

[bib17] Helgesson M., Björkenstam E., Rahman S., Gustafsson K., Taipale H., Tanskanen A. (2021). Labour market marginalisation in young adults diagnosed with attention-deficit hyperactivity disorder (ADHD): A population-based longitudinal cohort study in Sweden. Psychological Medicine.

[bib18] Helgesson M., Rahman S., Björkenstam E., Gustafsson K., Amin R., Taipale H. (2021). Trajectories of labour market marginalisation among young adults with newly diagnosed attention-deficit/hyperactivity disorder (ADHD). Epidemiology and Psychiatric Sciences.

[bib19] Helgesson M., Tinghog P., Niederkrotenthaler T., Saboonchi F., Mittendorfer-Rutz E. (2017). Labour-market marginalisation after mental disorders among young natives and immigrants living in Sweden. BMC Public Health.

[bib20] Holmlund L., Hellman T., Engblom M., Kwak L., Sandman L., Törnkvist L. (2022). Coordination of return-to-work for employees on sick leave due to common mental disorders: Facilitators and barriers. Disability & Rehabilitation.

[bib21] Kjeldgård L., Stigson H., Alexanderson K., Friberg E. (2020). Nov 16). Sequence analysis of sickness absence and disability pension in the year before and the three years following a bicycle crash; a nationwide longitudinal cohort study of 6353 injured individuals. BMC Public Health.

[bib22] Kupper T., Haavik J., Drexler H., Ramos-Quiroga J.A., Wermelskirchen D., Prutz C. (2012). The negative impact of attention-deficit/hyperactivity disorder on occupational health in adults and adolescents. International Archives of Occupational and Environmental Health.

[bib23] Lee C.S.C., Ma M.-T., Ho H.-Y., Tsang K.-K., Zheng Y.-Y., Wu Z.-Y. (2017). The effectiveness of mindfulness-based intervention in attention on individuals with ADHD: A systematic review. Hong Kong Journal of Occupational Therapy.

[bib24] Ludvigsson J.F., Andersson E., Ekbom A., Feychting M., Kim J.-L., Reuterwall C. (2011). External review and validation of the Swedish national inpatient register. BMC Public Health.

[bib25] Ludvigsson J.F., Svedberg P., Olen O., Bruze G., Neovius M. (2019). The longitudinal integrated database for health insurance and labour market studies (LISA) and its use in medical research. European Journal of Epidemiology.

[bib26] Mongia M., Hechtman L. (2012). Cognitive behavior Therapy for adults with attention-deficit/hyperactivity disorder: A review of recent randomized controlled trials. Current Psychiatry Reports.

[bib27] Murley C., Tinghög P., Karampampa K., Hillert J., Alexanderson K., Friberg E. (2020). Types of working-life sequences among people recently diagnosed with multiple sclerosis in Sweden: A nationwide register-based cohort study. BMJ Open.

[bib28] National Board of Health and Welfare (2014).

[bib29] National Board of Health and Welfare (2020). Bortfall och kvalitet i patientregistret (Loss and quality in the patient register. https://www.socialstyrelsen.se/statistik-och-data/register/alla-register/patientregistret/bortfall-och-kvalitet/.

[bib30] Robbins R. (2017). The untapped potential of the ADHD employee in the workplace. Cogent Business & Management.

[bib31] Rydell M., Lundström S., Gillberg C., Lichtenstein P., Larsson H. (2018). Has the attention deficit hyperactivity disorder phenotype become more common in children between 2004 and 2014? Trends over 10 years from a Swedish general population sample. Journal of Child Psychology and Psychiatry.

[bib32] Shaw W.S., Kristman V.L., Williams-Whitt K., Soklaridis S., Huang Y.H., Côté P. (2014). The job accommodation scale (JAS): Psychometric evaluation of a new measure of employer support for temporary job modifications. Journal of Occupational Rehabilitation.

[bib33] Swedish Social Insurance Agency (2012).

[bib34] Wettermark B., Hammar N., Fored C.M., Leimanis A., Otterblad Olausson P., Bergman U. (2007). The new Swedish Prescribed Drug Register--opportunities for pharmacoepidemiological research and experience from the first six months. Pharmacoepidemiology and Drug Safety.

[bib35] Zetterqvist J., Asherson P., Halldner L., Långström N., Larsson H. (2013). Stimulant and non‐stimulant attention deficit/hyperactivity disorder drug use: Total population study of trends and discontinuation patterns 2006–2009. Acta Psychiatrica Scandinavica.

