# Supplementary material for: Sustainable labour market participation among working young adults with diagnosed attention deficit/hyperactivity disorder (ADHD)
Source: SSM Popul Health. 2023 Jun 12;23:101444. doi: 10.1016/j.ssmph.2023.101444 (PMC10492158; doi:10.1016/j.ssmph.2023.101444)
Supplement: Multimedia component 1 [file mmc1.docx]

**Supplementary Table 1.** Measures of cluster partition quality for different number of clusters^a^ for those diagnosed with ADHD and controls, respectively.

|  | **PBC^b^** | **HG^c^** | **HGSD^d^** | **ASW^e^** | **ASW(w)^f^** | **R2^g^** | **R2sq^h^** | **HC^i^** |
| --- | --- | --- | --- | --- | --- | --- | --- | --- |
| **ADHD** |  |  |  |  |  |  |  |  |
| 4 Clusters | 0,74 | 0,89 | 0,89 | 0,43 | 0,43 | 0,41 | 0,63 | 0,05 |
| **5 Clusters** | **0,75** | **0,91** | **0,91** | **0,42** | **0,42** | **0,44** | **0,67** | **0,04** |
| 6 Clusters | 0,69 | 0,88 | 0,88 | 0,35 | 0,35 | 0,48 | 0,69 | 0,06 |
| 7 Clusters | 0,70 | 0,89 | 0,89 | 0,34 | 0,34 | 0,49 | 0,72 | 0,06 |
| 8 Clusters | 0,68 | 0,89 | 0,89 | 0,32 | 0,32 | 0,51 | 0,73 | 0,06 |

^a^ The selected number of clusters in bold text; ^b^ Point Biserial Correlation; ^c^ Hubert’s Gamma; ^d^ Hubert’s Somers’ D; ^e^ Average Silhouette Width; ^f^ Average Silhouette Width (weighted); ^g^ Pseudo R2; ^h^ Pseudo R2 squared;^i^ Hubert’s
